# Analyzing Information Disparities across Modalities in Mortality Prediction

**DOI:** 10.1101/2025.10.30.25339162

**Published:** 2025-11-02

**Authors:** Chanhwi Kim, WonJin Yoon, Hoonick Lee, Jung-Oh Lee, Majid Afshar, Jaewoo Kang, Timothy Miller

**Affiliations:** 1 Dept. of Computer Science and Engineering, Korea University, Seoul, 02841, Republic of Korea.; 2 Dept. of Pediatrics, Harvard Medical School, Boston, MA 10587, USA.; 3 Computational Health Information Program, Boston Children’s Hospital, Boston, MA 02115, USA.; 4 Aigen Sciences, Seoul, 04778, Republic of Korea.; 5 Dept. of Diagnostic, Molecular and Interventional Radiology, Icahn School of Medicine at Mount Sinai, New York, NY, 10029, USA.; 6 Dept. of Medicine, University of Wisconsin–Madison, Madison, WI 53706, USA.

**Keywords:** Post-discharge Mortality, Vision–Language Models, Multimodal Learning, Chest Radiographs, Electronic Health Records

## Abstract

Recent advances in deep learning have enabled the integration of heterogeneous data modalities for clinical prediction, allowing models to exploit complex information embedded within electronic health records (EHRs). Among these modalities, chest radiographs (CXRs) provide a rich source of visual information that can enhance patient outcome prediction for patients in the intensive care unit (ICU). However, the comparative impact of different CXR representations—raw images versus radiology reports—on predictive performance has not been systematically investigated. Such comparisons are essential for identifying the most informative modality and understanding how it complements other data sources. This study compares the predictive utility of raw CXRs versus radiology reports for 30-day post-discharge mortality prediction in ICU patients. We employed a Vision–Language Model (VLM) with patient discharge notes. On a filtered subset of the MIMIC-IV dataset (n = 1,360), augmenting discharge notes with CXRs achieved the best performance (AUROC = 0.843), surpassing both the discharge-note-only (AUROC = 0.816) and radiology-report-augmented (AUROC = 0.804) models. The experiments demonstrated that combining raw CXRs with discharge notes consistently outperformed models augmented with radiology reports. A radiologist’s review further revealed that reports often omitted clinically relevant findings visible in the images, highlighting that CXRs convey richer prognostic signals for mortality risk. These findings underscore the critical role of modality selection in clinical AI systems and suggest that textual summaries should be used as surrogates for multimodal data with caution, as they may fail to capture critical predictive information.

## 1 Introduction

Predicting patient outcomes after discharge from the intensive care unit (ICU) can be valuable for clinical decision-making, resource allocation, and improving patient outcomes [1–3]. Among possible patient outcomes, mortality prediction has received major attention from researchers because patients discharged from the ICU remain at elevated risk of death and readmission [3–7]. Electronic health records (EHRs), which contain both structured and unstructured data, have been widely utilized to address these risks. In particular, structured EHR data, such as demographics, vital signs, and laboratory results, has been successfully employed for mortality prediction [8–10]. Recent advances in natural language processing (NLP) and large language models (LLMs) have facilitated the extraction of prognostic signals from unstructured clinical narratives such as progress notes, discharge notes, and radiology reports [11, 12]. Among these clinical texts, discharge notes are particularly valuable for mortality prediction, as they provide comprehensive clinical information essential for post-discharge risk assessment [13]. Advanced clinical NLP models, such as BERT- and GPT-based models, can effectively process long textual contexts and capture clinically meaningful information that is often absent from structured data [14–17]. Building on these advances, models that jointly leverage structured and unstructured EHR data have achieved strong performance in predicting clinical outcomes [18, 19].

Beyond structured and unstructured EHR data, chest radiographs (CXRs) are also widely used to assess patient status and represent a rich source of prognostic information [20–22]. For example, CXRs can reveal clinically relevant features such as cardiomegaly, pleural effusion, and structural abnormalities. On the other hand, radiology reports, which are written by radiologists based on CXR examinations, typically summarize major findings or changes in a patient’s thoracic status. Consequently, CXRs and radiology reports have been integrated with structured EHR data to predict patient outcomes using machine learning and deep learning approaches [23, 24]. Some studies also incorporated unstructured clinical text, although most approaches have continued to rely on conventional deep learning pipelines [25, 26]. These efforts demonstrated that CXRs contain prognostic information beyond that available in both structured and unstructured EHR data. However, these studies have primarily focused on the performance gains achieved by incorporating CXRs and radiology reports, without addressing the differences in the information conveyed by themselves. To our knowledge, no previous work has applied vision–language models (VLMs) to CXR data alongside unstructured clinical text for mortality prediction while systematically comparing the predictive signal across modalities.

In this study, we aimed to investigate how each modality, including CXRs and radiology reports, contributes distinct information to patient outcome prediction. We examine three important research questions:

1. Can VLMs effectively combine CXRs with discharge note to improve the accuracy of post-discharge mortality prediction in the ICU?
2. Given that CXRs are almost always accompanied by radiology reports, are multimodal approaches necessary for integrating CXR-based information, or can text-only predictors based on radiology reports be equally effective?
3. What is the relative information content of different modalities for our mortality prediction task?

We defined 30-day post-discharge mortality as the primary endpoint and evaluated model performance using the Area Under ROC Curve (AUROC). By systematically evaluating CXRs and radiology reports alongside the discharge note, we demonstrate that different modalities capture distinct predictive signal relevant to mortality prediction. This study demonstrates that the choice of modality should be guided by the nature of the information to be conveyed to the model, underscoring that modality selection is a critical consideration in developing multi-modal clinical AI systems.

## 2 Results

This section systematically compares model performance for 30-day post-discharge mortality in ICU patients, quantified by AUROC, when discharge notes are augmented with CXRs or radiology reports. It also evaluates differences in information content across modalities employing Kendall’s τ-based distance (τ*-based distance*) and presents a post hoc radiologist analysis of discrepancies between CXRs and their corresponding radiology reports.

### 2.1 Main Results

**Fig. 1:**
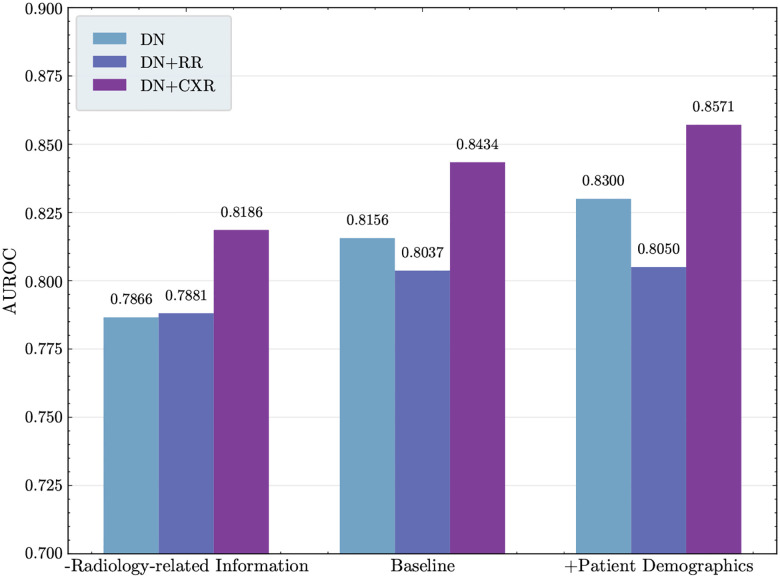
AUROC across input configurations. “Baseline” denotes discharge note summaries generated using three types of aspect: plain, risk factor, and timeline. “-Radiology-related Information” refers to discharge note summaries with radiology content removed during summarization, also produced in the same three aspect types “+Patient Demographics” corresponds to discharge note summaries augmented with patient demographics (age and race), likewise generated across the three aspect types. DN = discharge note summary, RR = radiology report, CXR = chest radiograph.

Figure 1 presents the AUROC values obtained under different input conditions (DN, DN+RR, and DN+CXR), where “DN” denotes discharge notes, “RR” denotes radiology reports, and “CXR” denotes CXRs, using the Llama-3.2-11B-Vision-Instruct model ^1^. Since the original discharge notes were too long for direct model finetuning, we adopted an aspect-oriented summarization approach that produced shortened inputs with focus on specific aspects of the note [27] and subsequently averaged the scores across three summarization aspects (plain, risk-factor, and timeline — described further in Methods).

When the model is trained solely on discharge note summaries (DN), the baseline summaries achieved an average AUROC of 0.8156. Incorporating radiology reports into these summaries (DN+RR) reduced performance to 0.8037. In contrast, combining CXRs with the summaries (DN+CXR) improved model performance, increasing the AUROC to 0.8434. To verify the statistical significance of these differences, we conducted DeLong’s tests. Compared with the DN experiments, all pairwise comparisons yielded p-values below 0.001, and compared with DN+RR, p-values were below 0.0001.

A similar trend was observed across alternative discharge note summary settings. Removal of the radiology-related information from the discharge note during summarization resulted in comparable AUROCs for the DN-only (0.7866) and DN+RR (0.7881) settings. In contrast, augmenting these summaries with CXRs improved the AUROC to 0.8186. To incorporate information that can be inferred from CXRs (age and race), we retrieved the corresponding patient demographics from the patient metadata and added them to the demographic section of each discharge note summary. This integration further improved predictive performance, yielding an AUROC of 0.8300. Adding radiology reports, however, did not yield additional benefit, with AUROC remaining close to the demographic-free setting (0.8050 vs. 0.8037). By contrast, combining CXRs with discharge note summaries and demographics markedly enhanced model’s performance, reaching an AUROC of 0.8571. This represents a gain of up to 5.2 percentage points compared with the radiology report–augmented setting (DN+RR, AUROC 0.8050).

Table 1 reports the detailed AUROC values for each summary aspect across different models. In this experiment, we evaluated three models with distinct architectures to assess the performance gains attributable to model design: (1) a Support Vector Machine (SVM) with TF-IDF(Term frequency-inverse document frequency)–weighted Bag-of-Words features, representing a traditional machine learning approach; (2) ModernBERT [28], an embedding-based model; and (3) the Llama-3.2-11B-Vision-Instruct, a vision-language model (VLM) capable of handling multimodal inputs. Both SVM and ModernBERT accept only textual inputs, they were evaluated in the DN and DN+RR settings.

As the SVM relies on a simple text-based representation, performance variations across different configurations were minimal. Although the model’s performance varied depending on the aspect of summarization, the plain summary consistently achieved the highest performance across all experimental settings, whereas the timeline aspect yielded the lowest.The average AUROC scores for the SVM ranged from 0.7425 to 0.7470.

ModernBERT consistently outperformed the SVM across all experimental settings, achieving an average AUROC of 0.8156 when trained solely on discharge note summaries. Removing the radiology information from the summaries led performance decline to 0.7866, while adding patient demographics improved performance to 0.8300. However, incorporating radiology reports into discharge note summaries resulted in only modest and inconsistent performance changes, whereas adding patient demographics led to a more noticeable decline.

Experiments with the Llama-3.2-11B-Vision-Instruct model demonstrated consistent performance improvements over the other models, highlighting the advantage of multi-modality for mortality prediction task. When the model was finetuned solely on discharge note summaries, the lowest AUROC was observed for the risk-factor summaries, whereas plain summaries consistently outperformed the other types. This trend persisted even when the radiology section was excluded or when patient demographics were appended. When radiology reports were incorporated with discharge notes, outcomes were mixed: in some cases, risk-factor summaries yielded the lowest AUROC, whereas in others, timeline summaries performed worst. The average AUROC also declined approximately 1–2 percentage points, compared with the discharge note-only setting, except when the radiology section had been removed from the summaries. By contrast, combining CXRs with discharge note summaries consistently produced the highest predictive performance across all input settings. In this configuration, timeline-focused summaries consistently produced the lowest AUROC.DeLong’s test for correlated ROC curves confirmed that the AUROC improvements achieved by the DN+CXR configuration were statistically significant compared with both DN and DN+RR models (p<0.001 across all summary types). Overall, incorporating CXRs into discharge note summaries using the VLM achieved the highest performance for mortality prediction.

### 2.2 Information differences between summaries and modalities

**Fig. 2:**
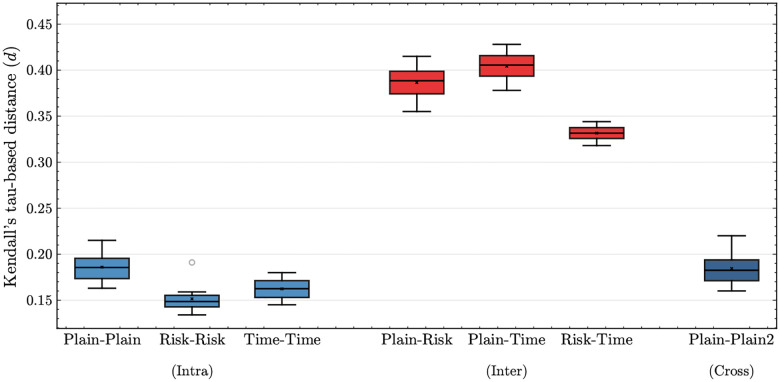
Distribution of τ*-based distances* among models trained with different discharge note summary types. “Intra” comparisons were computed between models trained on the same summarization aspect. “Inter” comparisons were computed by evaluating models trained on the different summarization aspect. “Cross” comparisons were computed between models trained on independently generated plain summaries (plain vs. plain2) derived from the same original discharge note.

**Fig. 3:**
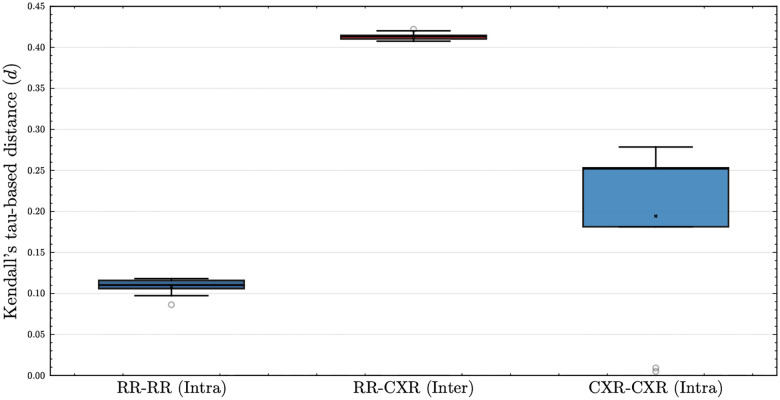
Distribution of τ*-based distances* across CXRs and radiology reports. “CXR” denotes CXRs and “RR” denotes radiology reports. “Intra” comparisons were computed between the model trained on the same modality. “Inter” comparisons were computed between the model trained on different modality.

We evaluated information complementarity across discharge note summarization aspects (Figure 2) and data modalities (Figure 3) to compare their differences. In these experiments, we followed the experimental setting and metric proposed by Yoon et al. [27]. The proposed metric, τ-*based distance*, takes values close to 0 when model-predicted rankings are highly similar, and approaches 0.5 when the rankings are largely uncorrelated.

Figure 2 demonstrated that aspect-oriented discharge note summaries capture distinct information. These comparisons were averaged across five models fintuned with different random seeds, using ModernBERT. “Intra” comparisons yielded lower τ-*based distance* (0.13–0.22) than “Inter” comparisons (0.32–0.43), indicating greater similarity of predicted rankings within aspects than across aspects. “Cross” comparisons produced τ-*based distances* of 0.16–0.23, which were comparable to those of “Intra” comparisons.

In Figure 3, we compared τ-*based distances* across modalities. We trained the Llama-3.2-11B-Vision-Instruct model to assess information differences between modalities. We observed that “Intra” comparisons within CXRs (0.18–0.28) and within radiology reports (0.09–0.13) yielded lower τ-*based distances* than “Inter” comparisons across CXRs and radiology reports (0.41–0.42). This reveals a similar pattern observed for aspect-oriented summarization of discharge note.

### 2.3 Post hoc analysis by radiologist

Table 2 presents the results of a radiologist’s post hoc review of 40 randomly selected pairs of CXRs and their corresponding radiology reports. The radiologist identified findings that were visible on the CXRs but not explicitly documented in the reports. These findings were categorized into anatomical and clinical groups for clarity. In the lung/diaphragm category, various unreported findings were observed, including pleural effusion, atelectasis, and emphysema. Many of these findings were documented only as “stable findings” without specific description. Additional omissions were noted across several other categories: skeletal degeneration (e.g., diffuse bony degeneration, osteopenia), mediastinal findings (e.g., aortic calcification, diverticulum with calcification), cardiomegaly, post-surgical findings (e.g., sternotomy wires, surgical clips), medical devices (e.g., NG tube, ET tube, EKG leads), and body habitus indicators (e.g., obesity, senile changes). While most unreported findings represent common age-related changes or routine medical devices that are frequently omitted from reports, some of these omissions were clinically relevant.

## 3 Discussion

Our study demonstrates that combining CXRs with discharge notes improves post-ICU mortality prediction compared with using discharge notes alone or when augmented with radiology reports. This finding confirms that CXRs provide complementary predictive signals that are not fully captured in text, even the radiology report text that nominally is a description of the CXR. Quantitative τ-*based distance* analyses and expert review further support this observation. Together, these results highlight that modality selection is critical in designing clinical prediction models, as performance depends not only on the content of information but also on the modality through which it is represented.

Building on this finding, we examined how different modeling approaches exploit these signals. Despite its simplicity, the SVM achieved strong performance, likely because discharge note summaries are lengthy and contain rich clinical information. Incorporating additional information, such as demographics or radiology reports, yielded only modest improvements, as these supplementary inputs constitute only a marginal portion of the overall content in the summaries. More advanced architectures pretrained on large-scale text corpora and capable of understanding contextual relationships showed clearer gains over SVM. ModernBERT consistently outperformed SVM across all experimental settings, with particularly notable improvements when patient demographics were included. Replacing ModernBERT with the Llama-3.2-11B-Vision-Instruct model produced an additional 1 percentage point increase in average of AUROC, even when limited to text data. Moreover, incorporating CXRs further improved AUROC by more than 4% on average compared with the discharge note–only setting with ModernBERT. These findings demonstrate that VLMs outperform their competitors even when used solely for text processing, and that leveraging additional modalities yields further gains by capturing complementary predictive information. Overall, models pretrained on large and diverse corpora effectively leverage knowledge between demographic variables and mortality risk. In addition, vision–language models jointly trained on text and image data offer distinct advantages for clinical prediction tasks, particularly when imaging modalities provide critical predictive signals.

Having established these architectural differences, the next question concerns how CXR-derived information itself should be represented. Removing the radiology-related information from discharge note summaries led to a notable decline in performance, underscoring the prognostic importance of CXR-derived content. These radiology sections integrate findings from multiple CXR studies obtained during an ICU stay, and their absence eliminates key predictive cues. Although removing the radiology section led to a decrease in performance, incorporating CXR resulted in an AUROC comparable to that obtained with the baseline discharge note summaries. This suggests that the information contained in a single CXR is roughly equivalent to that conveyed by the entire radiology section of a discharge note. These observations highlight the central role of CXR-derived information in outcome prediction, whether represented as raw images or summarized in textual reports. By contrast, augmenting selected patient demographic attributes (age and race) to the discharge note summaries improved model performance. However, because such variables can often be inferred directly from imaging, their contribution overlaps with imaging-derived information. Nevertheless, that they still provided additive gains suggests that CXRs encode signals distinct from demographic data. This reinforces the view that CXRs provide unique and clinically meaningful information not captured in textual reports or basic demographic variables.

Comparing radiology reports with CXRs further revealed important contrasts. Radiology reports, when added to discharge note summaries, reduced overall AUROC, except in the setting where the discharge notes’ radiology section was absent. On the other hand, adding CXRs improved performance across all experimental settings. These results reflect that including radiology reports may mislead the model by introducing redundancy with the radiology section of the discharge note summaries or by overemphasizing a single report. In contrast, a single CXR alone can provide additional predictive signals that enhance mortality prediction beyond what radiology reports can offer.

This interpretation is further supported by the τ-*based distance* analysis. τ-*based distance* was first validated by comparing “Intra”- and “Inter”-aspect discharge note summaries, replicating the findings of Yoon et al. [27] that aspect-oriented notes capture distinct subsets of information. In addition to the pre-designed experimental setting, we added “Cross” comparisons to validate the effect of random variation introduced across multiple summarizations of the same input. As we expected, we found that “Intra” comparisons showed lower τ-*based distance* than “Inter”-aspect. Moreover, “Cross” comparisons confirmed that this random variation does not substantially modify the underlying information of the original discharge notes when summarized. Extending the analysis to multimodal data revealed the same pattern: “Intra”-modality comparisons (within CXRs or within radiology reports) yielded lower τ-*based distances* than “Inter”-modality comparisons (across images and reports), indicating that CXRs and radiology reports contain distinct information. Also, the broader distribution of τ-*based distances* for CXR “Intra” comparisons suggests that CXRs could capture a wider, though less consistent, range of predictive signals. Taken together, these findings provide systematic evidence that CXRs and radiology reports encode substantially different information relevant to mortality prediction, demonstrating the utility of τ-*based distance* for multimodal evaluation.

A post hoc qualitative review by a radiologist supported these observations from a clinical perspective. The analysis revealed that certain clinical signals, such as small nodules, mild cardiomegaly, or subtle pleural effusions, were occasionally omitted from radiology reports. Severe cardiomegaly, diffuse degenerative skeletal changes, and evidence of emphysema were also underreported, even though these findings carry prognostic significance for post-discharge outcomes. Interestingly, general indicators of patient body habitus including obesity were missed in reports, although they may indirectly signal chronic disease burden or frailty. In addition, several readily identifiable devices, including the presence of endotracheal, nasogastric tubes, EKG leads, or air masks, were often excluded despite their clinical relevance in reflecting patient acuity or recent interventions. These omissions likely occurred because the analysis was based on the final CXRs obtained before discharge. When such findings in CXR were either stable or less pronounced compared with prior examinations, and then it might not be re-documented. Although radiology reports accurately summarize findings, they are guided by the ordering clinician’s indications and do not comprehensively describe all observable abnormalities visible on the images. As a result, those reports may fail to preserve all clinical cues from raw CXRs that are relevant for predictive modeling [29–32].

The collective evidence indicates that CXRs contain distinct and clinically meaningful signals that are not fully captured by textual data, such as discharge note summaries or demographic variables. While paired CXRs and radiology reports are valuable for model alignment, the narrative reports are not primarily designed for predictive tasks and may omit subtle but clinically significant cues present in the source image. Consequently, the direct integration of raw imaging data with clinical notes enables models to capture a more comprehensive spectrum of prognostic information. This underscores a critical principle for developing clinical AI: predictive performance is maximized by leveraging diverse, minimally processed multimodal inputs that preserve the inherent rich information of the raw data.

Despite these advancements, this study has limitations. This study was conducted using data from a single institution, which may limit the generalizability of the findings. We evaluated a general-purpose vision–language model rather than models specifically finetuned on clinical data. Currently, no VLMs have been pretrained using multimodal data for mortality prediction tasks that jointly utilize discharge notes and CXRs; therefore, we leveraged a well-aligned general-purpose VLM instead. In addition, only the most recent CXR per patient was given to model, as none of the available VLMs are trained on interleaved or temporally ordered CXR sequences. This architectural constraint restricted our analysis precluding the ability to capture temporal changes in clinical information that can be observed across serial CXRs or multiple views. Future investigations that leverage multi-institutional cohorts and models pretrained on large-scale datasets of discharge notes and CXRs, and that are capable of processing longitudinal imaging sequences, will be essential to validate and extend these findings.

## 4 Methods

This study employed a multimodal methodological framework integrating discharge notes, radiology reports, and CXRs to predict post-ICU patient mortality. This section details the dataset construction, preprocessing steps, summarization strategies, model architectures, training and evaluation procedures used in our experiments.

### 4.1 Tasks of interest

The primary task in this study is 30-day post-discharge mortality prediction for ICU patients, which following the definition of Yoon et al. [27]. It is formulated as a binary classification problem where the objective is to predict whether a patient will die within 30 days after ICU discharge (y∈{0,1}). Unlike structured severity scores or coded diagnoses, this task emphasizes prediction based on unstructured clinical narratives and raw imaging data, thereby enabling an evaluation of multimodal representations for predictive modeling.

### 4.2 Dataset

#### 4.2.1 Overall dataset

In this study, we constructed a cohort by integrating textual and imaging data from two publicly available sources. The discharge notes were obtained from the LCD benchmark dataset [33], which is derived from MIMIC-IV [34], while the corresponding radiology reports and CXRs were retrieved from MIMIC-IV-CXR [35, 36]. To ensure consistency across modalities, we restricted the cohort to patients who had at least one CXR study during their ICU stay.

The textual inputs consist of de-identified discharge notes and radiology reports associated with ICU admissions at the Beth Israel Deaconess Medical Center (2009–2019), linked to out-of-hospital mortality outcomes from the Massachusetts State Registry of Vital Records and Statistics. After filtering, the final dataset contained 6,345 patients in the training set (from 34,758 originally), 1,367 patients in the development set (from 7,505), and 1,360 patients in the test set (from 7,568). Appendix B summarizes the distribution of studies and CXRs per patient, and Appendix C provides statistics on discharge note token lengths.

When multiple CXR studies were available for a single patient, we selected the image that best represented the patient’s clinical status. Ideally, leveraging all available CXRs would better capture longitudinal changes in patient status, However, this approach was limited by (i) the large variability in the number of chest radiographs (ranging from 1 to more than 30) and (ii) the lack of validated models capable of handling long, interleaved multi-image sequences. To address this, a heuristic decision tree which is developed with physician was used. Each image was assigned a priority score based on procedure type, view, and orientation; lower scores indicated higher priority (unmapped values defaulted to 99). For instance, *posteroanterior (PA)* views were preferred over *anteroposterior (AP)* views, and standard images were prioritized over portable ones.

This prioritization scheme ensured that the model received images captured under comparable conditions, thereby facilitating consistent feature extraction and reflected typical clinical practice. PA images are typically acquired with the patient standing and in a relatively stable condition, whereas AP or portable images are often obtained when the patient is unable to sit or stand due to illness severity. Accordingly, the algorithm preferentially selected studies that best represented each patient’s physiological status under more normal conditions. Within each patient’s ICU stay, images in the highest-priority group were ordered chronologically, and the most recent image was chosen to represent the patient’s best condition. All selected CXRs were resized to 512 × 512 pixels for computational efficiency. The pseudocode for the selection procedure is provided in Appendix E.

#### 4.2.2 Aspect-orient summarization of discharge notes for finetuning

To mitigate the computational cost associated with finetuning on full-length discharge notes, we employed the aspect-oriented summarization framework proposed by Yoon et al. [27]. Each discharge note was summarized using the Llama-3.2-11B-Vision-Instruct model, with prompts tailored to predefined clinical aspects: (1) plain summary, (2) risk factor-focused summary, and (3) timeline-focused summary. During baseline summarization, the model temperature was set to 0.1, following prior work indicating that lower temperatures produce more consistent and reliable summaries [37]. To remove radiology information from the discharge note, the model was prompted to generate summaries that excluded CXR-related information from the original discharge notes. The generated outputs were quality-checked through keyword filtering, manual sampling, and post-processing when necessary. To incorporate selected patient demographics, we converted each patient’s age and race into text form and appended them to the personal information section of the discharge note summaries. On average, this summarization procedure reduced the token count by approximately 75% compared with the original discharge notes (see Appendix C). The detailed prompts used for summarization are provided in Appendix A.

### 4.3 Baseline model selection

We compared three types of models: a traditional machine learning approach, an embedding-based model, and a vision–language model (VLM). The SVM was used as the representative of traditional machine learning, chosen for its robustness and interpretability in small-sample, high-dimensional clinical text settings. Implemented with the scikit-learn library [38], the model was trained on TF-IDF–weighted Bag-of-Words features derived from discharge note summaries and radiology report texts. For the embedding-based category, we employed ModernBERT [28], the most recent model capable of processing long-context inputs (up to 8,192 tokens), allowing full utilization of discharge note summaries. For the VLM category, we evaluated three different candidate models (Llama-3.2-11B-Vision-Instruct, Phi-3.5-Vision-Instruct, and Pixtral-12B), aiming to identify a model capable of capturing clinical signals from discharge note and visual inputs We used the Hugging Face Transformers library [39] to load pretrained weights and finetuned the transformer-based models. Among these candidates, the Llama-3.2-11B-Vision-Instruct model achieved the highest zero-shot F1 score than the other candidates (Table 3), therefore selected as the baseline VLM. Yet, its performance decreased upon incorporating CXRs, suggesting that the model effectively leverages textual information but fails to capture prognostic signals from radiographic inputs. To ensure consistent comparison across VLMs, zero-shot experiments were conducted without a classification head, allowing each model to directly predict binary outcomes (0: alive, 1: dead). Because probability estimates were unavailable, performance was evaluated using the F1 score, with the temperature fixed at 0 for deterministic outputs. Comparisons across VLMs were based on plain discharge note summaries.

### 4.4 Evaluation metric

We evaluated model performance using the *Area Under the Receiver Operating Characteristic curve* (AUROC) and the τ-*based distance*. AUROC summarizes threshold-free ranking performance as the area under the ROC curve, equal to the probability that a random positive is scored above a random negative. To capture informational differences between models, we employed *Kendall’s tau coefficient (τ)* [40], which quantifies the rank correlation between two sets of predictions. Let $L_{1}=\left\{p_{1}^{1},p_{2}^{1},\ldots ,p_{k}^{1}\right\}$ and $L_{2}=\left\{p_{1}^{2},p_{2}^{2},\ldots ,p_{k}^{2}\right\}$ be the probability outputs of two models $M_{1}$ and $M_{2}$ on the same set of k notes, and let $R_{1}$ and $R_{2}$ be the corresponding rank lists. Define

$$N=\frac{k(k-1)}{2},$$

P=#{concordantpairs},

Q=#{discordantpairs},

$$T=\#\left\{\text{tied}\;\text{pairs}\;\text{in}\;R_{1}\right\},$$

$$U=\#\left\{\text{tied}\;\text{pairs}\;\text{in}\;R_{2}\right\}.$$

Following Kendall [40], the Kendall’s tau coefficient between $L_{1}$ and $L_{2}$ is defined as

(1) $$\tau_{1,2}=\frac{P-Q}{\sqrt{\left(N-T)(N-U\right)}}.$$

The coefficient τ takes values in [−1, 1], where τ=-1 indicates complete reversal of the rankings, τ=0 corresponds to random or uncorrelated rankings, and τ=1 denotes identical rankings. Following Yoon et al. [27], we then defined τ-*based distance*

$$d_{i,j}=\frac{1-\tau_{i,j}}{2},\;d_{i,j}\in \left[0,1\right],$$

such that d=0 indicates perfect agreement, d=0.5 random correlation, and d=1 complete disagreement. We computed three types of distances:

- *“Intra”-summary distances*: distances between prediction rankings obtained from models trained on the same aspect-oriented summary type.
- *“Inter”-summary distances*: distances between prediction rankings from models trained on different aspect-oriented summary type.
- *“Cross”-summary distances*: distances between prediction rankings from models trained on different summary with same aspect-oriented summary type. We defined plain 2 as re-generated plain summary from same discharge note.

The “Cross”-summary distance was newly introduced in this study, as it was not evaluated in prior work [27], was designed to isolate stochastic effects during the summarization. To assess the effect of random initialization during finetuning, we trained n models $M_{1},\ldots ,M_{n}$ with different seeds (n=5). For each pair of models $\left(M_{i},M_{j}\right)$, we computed the τ-*based distance* $d_{i,j}$ between their predicted rankings on the same evaluation set.

### 4.5 Finetuning and Evaluation

We adopted the Low-Rank Adaptation (LoRA) technique [41] to enable computationally efficient finetuning. LoRA updates only a small number of low-rank adaptation matrices while keeping the pretrained model parameters frozen, thereby reducing memory requirements and training cost without compromising performance. The adapters were applied to all submodules, including the vision encoder, multimodal projector, and language model, and a classification head was attached to the final representation. Training was performed on the training set, hyperparameters were selected based on performance on the development set, and final evaluation was conducted on the test set. All datasets followed identical preprocessing across training and evaluation step to ensure consistency. During inference, the temperature was fixed at 0 to produce deterministic predictions and reduce stochastic variation. Detailed hyperparameter configurations are provided in Appendix D.

### 4.6 Post-hoc image analysis

For the post-hoc qualitative analysis of chest radiographs, a radiology expert was given paired CXRs and radiology reports. The radiologist was asked to note any visible phenomena in the images that might be useful for predicting mortality but were not described in the corresponding radiology reports. We then categorized the findings according to their clinical relevance.

### 4.7 Ethical considerations

This study utilized data from MIMIC-IV database [34–36], including de-identified discharge note, CXRs, and radiology reports. All data have undergone rigorous deidentification in accordance with the HIPAA Safe Harbor standards, as documented in Johnson et al. [34]. Protected health information (PHI) was removed through date shifting, name redaction, and free-text masking using both rule-based and neural network–based methods. In the discharge summaries, no explicit PHI remains; temporal fields (e.g., admission, discharge, and birth or death dates) were uniformly shifted to future years (2100–2200) while preserving relative temporal intervals. In certain experimental settings, we incorporated patient age and race information by referencing the MIMIC-IV mapping tables, which were also used to link CXRs with their corresponding radiology reports and discharge note. However, all data processing and analysis procedures were conducted strictly for research purposes, in full compliance with the MIMIC data use agreement and applicable ethical standards, with no attempt made to re-identify any individual patient.

## Figures and Tables

**Table 1:** AUROC performance across experimental conditions.

Model	SVM		ModernBERT		LLaMA 3.2-11B		
Summary Type	DN	DN+RR	DN	DN+RR	DN (p-value)	DN+RR (p-value)	DN+CXR (ref)
- *Radiology-related Information*							
Plain	0.7527	0.7659	0.7962	0.7831	0.8263 (p=3.8e-5)	0.8121 (p=2.5e-5)	**0.8300**
Risk Factor	0.7419	0.7574	0.8080	0.8105	0.7659 (p=1.1e-5)	0.7724 (p=2.3e-6)	**0.8159**
Timeline	0.7330	0.7075	0.7450	0.7590	0.7675 (p=1.6e-3)	0.7799 (p=1.6e-7)	**0.8099**
*Average*	0.7425	0.7436	0.7830	0.7842	0.7866	0.7881	**0.8186**
*Baseline*							
Plain	0.7868	0.7963	0.8219	0.8325	0.8392 (p=1.7e-6)	0.8261 (p=1.4e-6)	**0.8597**
Risk Factor	0.7466	0.7419	0.7920	0.7793	0.8001 (p=3.4e-4)	0.7978 (p=5.8e-5)	**0.8374**
Timeline	0.7072	0.7012	0.7911	0.7872	0.8076 (p=5.4e-6)	0.7873 (p=2.9e-7)	**0.8332**
*Average*	0.7469	0.7465	0.8017	0.7997	0.8156	0.8037	**0.8434**
*+ Patient Demographics*							
Plain	0.7872	0.7962	0.8282	0.8329	0.8414 (p=4.2e-5)	0.8120 (p=5.8e-6)	**0.8657**
Risk Factor	0.7467	0.7419	0.8166	0.7894	0.8164 (p=2.9e-6)	0.8032 (p=1.4e-7)	**0.8535**
Timeline	0.7072	0.7012	0.8057	0.7614	0.8323 (p=2.3e-6)	0.7998 (p=7.1e-8)	**0.8522**
*Average*	0.7470	0.7464	0.8168	0.7946	0.8300	0.8050	**0.8571**

p-values were computed using DeLong’s test for correlated ROC curves, with DN+CXR serving as the reference model. LLaMA 3.2-11B-vision-instruct is abbreviated as LLaMA 3.2-11B. DN: discharge note summary; RR: radiology report; CXR: chest radiograph. Bold indicates the best performance in each row.

**Table 2:** The categorized findings refer to abnormalities that were present on the CXRs that were identified by expert radiologists but not explicitly documented in the official radiology reports.

Category	Findings not documented in the radiology report
Lung / Diaphragm	Coarse lung parenchyma, diaphragmatic flattening, retrocardiac opacity, mild atelectasis, emphysema, pleural effusion
Mediastinum	Aortic calcification/atherosclerosis, unfolded aorta, esophageal diverticulum with calcification, pneumomediastinum
Skeletal degeneration	Diffuse bony degeneration, shoulder arthrosis, costochondral junction calcification, osteopenia
Heart	Cardiomegaly
Post-surgical findings	Sternotomy wire, surgical clips (CABG)
Devices	NG tube, ET tube, chest tube, CVC, EKG leads/electrodes, facial/air mask
Body habitus	Senile changes (skin wrinkling), young skeletal appearance, obesity, cachexia
Other	Poor positioning

These findings were observed in the random subset of 40 cases. While some may be clinically relevant, many represent common findings that are frequently omitted from reports, including age-related changes, degenerative findings, routine medical devices, or abnormalities noted only as ”stable” without specific description. Abbreviations: CABG = coronary artery bypass graft; NG = nasogastric; ET = endotracheal; CVC = central venous catheter; EKG = electrocardiogram

**Table 3:** F1 score of baseline candidate models.

Model	DN	DN+CXR
Llama-3.2-11B-Vision-Instruct	**0.3502**	**0.3389**
Phi-3.5-vision-instruct	0.1809	0.1479
Pixtral-12B	0.1005	0.0989

DN: discharge note summary; CXR: chest radiograph.

**Fig. B:**
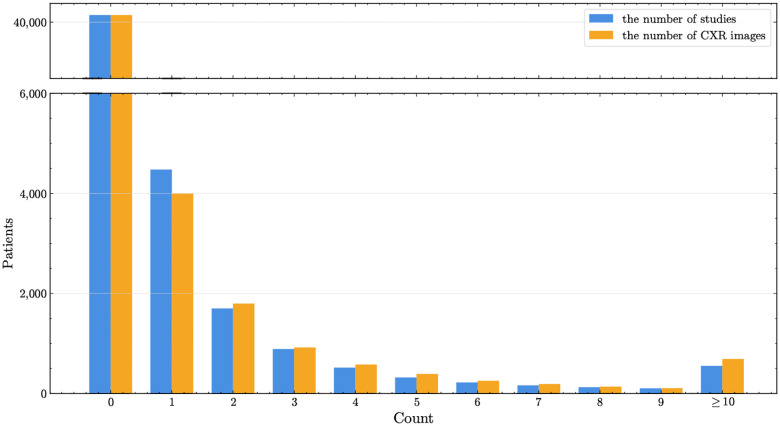
Number of patients grouped by the number of studies and CXRs obtained during the ICU stay. The graph presents aggregated counts across all splits (Train, Dev, and Test), and each split has a similar distribution.

**Fig. C:**
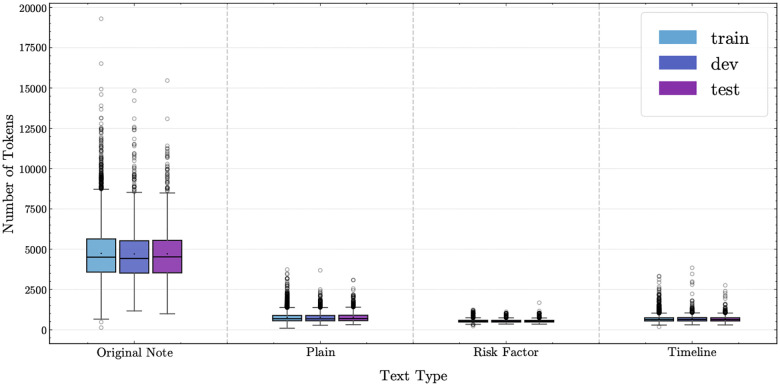
Distribution of token lengths for the original discharge notes and their summaries. Each data split (Train, Dev, and Test) demonstrates a similar distribution.

## Data Availability

The data underlying this article were derived from the LCD benchmark: https://github.com/Machine-Learning-for-Medical-Language/long-clinical-doc. Also, MIMIC-CXR datasets were derived from sources: https://physionet.org/content/mimic-cxr/2.1.0/ and https://physionet.org/content/mimic-cxr-jpg/2.1.0/.

## Footnotes

1
https://huggingface.co/meta-llama/Llama-3.2-11B-Vision-Instruct

## Code availability:

Preprocessing and training Code will be open-sourced.

## A Prompt

### Summary Prompt

System Prompt:

You are a highly trained medical assistant specializing in clinical documentation and summarization. Your task is to produce concise, accurate summaries of discharge notes, relying only on the information explicitly provided in the original document. Omit any personally identifiable details (e.g., names, ages, races, or ID numbers), and focus strictly on clinically relevant information.

Plain Summary Prompt:

Here is the clinical discharge document: {DISCHARGE NOTE} Please generate a summary of overall ICU discharge note that includes important clinical information. Write the summary as concise, clear, and well-organized bullet points.

Risk factor Summary Prompt:

Here is the clinical discharge document: {DISCHARGE NOTE}

Please generate a summary of potential risk factors from the discharge note, including relevant details with a balanced perspective by summarizing both risk factors and signs of stability or a positive prognosis, outlining:

- Clinical Stability: The patient’s current physical stability and any lingering critical conditions.
- Past History: The patient’s medical history and any pre-existing conditions that could influence recovery.
- New Diagnosis: Any new diagnoses made during the patient’s stay.
- Follow-Up: The discharge instructions and any recommended outpatient care.
- Adherence: Any notes or concerns regarding the patient’s ability to follow treatment plans.
- Substance Use: Any documented use of drugs, alcohol, or smoking.
- Other Factors: Any additional risks or notable circumstances exAplicitly mentioned in the discharge note.

Please present your summary in concise, clear, and well-organized bullet points.

Timeline Summary Prompt:

Here is the clinical discharge document: {DISCHARGE NOTE}

Please generate a timeline summary of the discharge note, outlining:

- Chronological Progression: The chronological progression of the patient’s ICU stay from admission to discharge.
- Significant Clinical Changes: Significant changes in the patient’s clinical condition over time.
- Timeline of Interventions: The timeline of interventions specific to the ICU setting.
- Notable Complications or Setbacks: Any notable complications or setbacks encountered during the ICU stay.
- Changes in Care Plans: Modifications to the patient’s care plan or treatment approach.

Please present your summary in concise, clear, and well-organized bullet points.

\section{Inference Prompt}\label{inference_prompt}

### Inference Prompt

Discharge note only

System Prompt: Below is a clinical discharge document. Based on the given clinical context, assess how likely the patient’s out-of-hospital mortality is within 30 days.

User Prompt: Here is the clinical discharge document: {DISCHARGE NOTE} Based on the clinical information provided, how likely is the patient’s out-of-hospital mortality within 30 days? Please do not explain the reason and respond with one word only: 0:alive, 1:death. Assistant:

CXR only

System Prompt: A single, most recent chest radiograph (CXR) from the patient is provided. Based on the provided CXR, assess how likely the patient’s out-of-hospital mortality is within 30 days.

User Prompt: {CXR} Based on the provided single CXR, how likely is the patient’s out-of-hospital mortality within 30 days? Please do not explain the reason and respond with one word only: 0:alive, 1:death. Assistant :

Radiology report only

System Prompt: A most recent radiology report from the patient is provided. Based on the provided radiology report, assess how likely the patient’s out-of-hospital mortality is within 30 days.

User Prompt: Here is the radiology report: {RADIOLOGY REPORT} Based on the radiology report provided, how likely is the patient’s out-of-hospital mortality within 30 days? Please do not explain the reason and respond with one word only: 0:alive, 1:death. Assistant:

Discharge note + Radiology report

System Prompt: A clinical document and a most recent radiology report are provided. Based on the clinical context and the radiology report, assess how likely the patient’s out-of-hospital mortality is within 30 days.

User Prompt: Here is the clinical discharge document: {DISCHARGE NOTE} Here is the radiology report: {RADIOLOGY REPORT} Based on the provided clinical information and radiology report, how likely is the patient’s out-of-hospital mortality within 30 days? Please do not explain the reason and respond with one word only: 0:alive, 1:death. Assistant:

Discharge note + CXR

System Prompt: A clinical document and a single, most recent chest radiograph (CXR) from the patient are provided. Based on the clinical context and the provided CXR, assess how likely the patient’s out-of-hospital mortality is within 30 days.

User Prompt: {CXR} Here is the clinical discharge document: {DISCHARGE NOTE} Based on the provided clinical information and single CXR, how likely is the patient’s out-of-hospital mortality within 30 days? Please do not explain the reason and respond with one word only: 0:alive, 1:death. Assistant:

## D Training hyperparameters

**Table D:** Hyperparameter search space and selected values for each model and input configuration.

Hyperparameter	Search space	DN	DN+RR	DN+CXR
**SVM**				
Kernel	[linear, rbf]	rbf	rbf	–
C	[0.1, 1, 10,]	1	1	–
Gamma	[scale, auto]	auto	auto	–
Feature dimension	[5000]	5000	5000	–
**Mo dernBERT**				
Learning rate	[2e-5, 1e-5, 5e-6]	1e-5	1e-5	–
Batch size	[8, 16]	8	8	–
Epochs	[10, 20, 30]	20	20	–
**LLaMA 3.2-11B-vision-instruct**				
Learning rate	[2e-5, 1e-5, 5e-6, 2e-6, 1e-6]	1e-6	2e-6	2e-6
Batch size	[8, 16]	16	16	16
Epochs	[10, 20, 30]	30	30	30

DN = discharge note, RR = radiology report, CXRs = chest radiographs.

## E CXR Selection Decision Tree

**Algorithm 1:** Selection algorithm for optimal chest radiographs based on hierarchical prioritization.

1:	**Define Priority Mappings:**
2:	*ProcedurePriority* ← {
	CHEST(PA AND LAT) → 1,
	CHEST(PRE-0P PA AND LAT) → 1,
	CHEST(PA AND LAT PORT) → 2,
	CHEST(P0RTABLE AP) → 3,
	…
	OTHER → 99
3:	}
4:	*ViewPriority* ← {
	PA → 1,
	AP → 2,
	LATERAL → 3,
	OTHER → 99
5:	}
6:	*OrientationPriority* ← {
	Erect → 1,
	Recumbent → 2,
	OTHER → 99
7:	}
8:	**function** SelectBestCXR(𝒟)
9:	**if 𝒟=∅ then**
10:	**return** null
11:	**end if**
12:	**for** each x∈𝒟 **do**
13:	p←ProcedurePriority[x.procedure]
14:	v←ViewPriority[x.view]
15:	o←OrientationPriority[x.orientation]
16:	x.priority←(p,v,o)
17:	**end for**
18:	$p^{*}\leftarrow {\text{min}}_{x\in 𝒟}\;\text{x.priority}$
19:	$𝒞\leftarrow \left\{x\in 𝒟:\text{x.priority}=p^{*}\right\}$
20:	**return** ${\text{argmax}}_{x\in 𝒞}\text{timestamp}(x)$
21:	**end function**
